# Changes of hormone levels for postmenopausal women after bilateral oophorectomy: A meta-analysis

**DOI:** 10.1097/MD.0000000000043726

**Published:** 2025-08-08

**Authors:** Xiaoqin Wu, Yong Lin, Yan Long, Qinqin Yi

**Affiliations:** a Luzhou Maternal and Child Health Hospital (Luzhou Second People’s Hospital), Sichuan Province, China.

**Keywords:** estradiol, hormone, meta-analysis, oophorectomy, postmenopausal, testosterone

## Abstract

**Background:**

Bilateral oophorectomy has a significant effect on changes in hormone levels in postmenopausal women. This meta-analysis aimed to evaluate the effects of bilateral oophorectomy on estradiol, testosterone, androstenedione, dehydroepiandrosterone sulfate (DHEAS), dehydroepiandrosterone (DHEA), sex hormone-binding globulin (SHBG), and estrone levels.

**Method:**

We conducted a search for studies on focused ultrasound for cervical high-grade squamous intraepithelial lesions in PubMed, EMBASE, Web of Science, and Cochrane Library databases. This review included 7 studies related to bilateral ovariectomy or retention in postmenopausal women. Using Stata software version 12.0, we applied the random effect model, fixed effect model, and subgroup analysis to evaluate the change of different hormones.

**Results:**

After bilateral ovariectomy, estradiol levels decreased significantly (standardized mean difference [SMD] = −0.26, 95% confidence interval [CI] [−0.50, −0.02], *P* = .031). The overall analysis did not show significant differences (SMD = −0.10, 95% CI [−0.46, 0.26], *P* = .585), although significant differences were observed between subgroups (*P* = .025). Testosterone levels also decreased significantly after bilateral ovariectomy (SMD = −0.58, 95% CI [−0.86, −0.31], *P* < .001). The overall analysis indicated a significant difference between the 2 groups (SMD = −0.35, 95% CI [−0.63, −0.07], *P* = .014). DHEA levels decreased significantly after bilateral ovariectomy (SMD = −0.51, 95% CI [−0.93, −0.10], *P* = .015). In contrast, the hormone levels of androstenedione, DHEAS, SHBG, and estrone did not show significant differences between the 2 groups: androstenedione (SMD = −0.04, *P* = .682), DHEAS (SMD = −0.07, *P* = .489), SHBG (SMD = −0.02, *P* = .781), and estrone (SMD = −0.04, *P* = .587).

**Conclusions:**

The present meta-analysis showed that bilateral ovariectomy had a significant effect on both estrogen and androgen levels in postmenopausal women, especially estradiol, testosterone, and DHEA. In contrast, the changes in androstenedione, DHEAS, SHBG, and estrone were not obvious. The findings of this study underscore the importance of monitoring hormone levels in postmenopausal women in clinical practice and considering the impact of oophorectomy on women’s long-term health when developing individualized treatment options.

## 1. Introduction

Menopause refers to a natural physiological process that a woman goes through. It usually occurs between the ages of 40 and 60. It is defined as the absence of menstrual bleeding in women after a period of 12 months, marking the end of fertility, and is associated with gradual decline in ovarian function and lower estrogen levels. Postmenopausal women refer to women who have gone through menopause. With the increase in average life expectancy, the postmenopausal period for women tends to extend. The debate over whether to remove healthy ovaries during this time remains contentious.

One perspective suggests that postmenopausal ovaries are unnecessary, as they are believed to cease hormone production. This viewpoint has resulted in the preventive removal of healthy ovaries during hysterectomy in postmenopausal women, with the intention of reducing the risk of ovarian cancer, even among those considered to be at low risk for ovarian cancer.

Another perspective suggests that postmenopausal ovaries retain endocrine activity, which contributes to the maintenance of androgen hormone levels essential for cardiovascular health,^[1]^ sexual function,^[2]^ and bone mineral density.^[3]^ Several observational studies have indicated that undergoing bilateral oophorectomy before reaching postmenopause can adversely affect the health of postmenopausal women. This impact includes an increased incidence of cardiovascular disease,^[4]^ osteoporosis,^[5]^ neurological disorders,^[6]^ depression,^[7]^ and changes in sexual function.^[8]^ Therefore, during gynecological surgery for postmenopausal women, if the ovaries are healthy, they should be preserved.

So far, no relevant meta-analysis has confirmed these perspectives or clarified the extent of the effect of oophorectomy on sex hormone levels after menopause. In this study, a meta-analysis was conducted to compare the effects of bilateral oophorectomy on hormone levels in postmenopausal women. The aim of the study is to provide reference and evidence-based medical data for clinical diagnosis and treatment by evaluating the impact of bilateral oophorectomy on hormone levels in postmenopausal women.

## 2. Materials and methods

### 2.1. Inclusion and exclusion criteria

*Inclusion criteria*: (1) randomized controlled trials (RCTs), observational studies, comparative studies, clinical trials, clinical studies, multicenter studies, and controlled studies. (2) The subjects must be postmenopausal women. Outcome measures should include the mean and standard deviation values of estradiol, testosterone, and other relevant hormones. (3) The literature is limited to publications in the English language. *Exclusion criteria*: (1) animal experiments, cellular studies, reviews, meta-analyses, case reports, or letters to the editor. (2) Duplicate reports of the same data. (3) Literature containing erroneous data. (4) Studies with outcome measures that do not meet the inclusion criteria or that contain significant errors or omissions.

### 2.2. Search strategy

To identify eligible studies, we conducted a comprehensive search of PubMed, Embase, Web of Science, and the Cochrane Library from the inception of these databases until November 2024. The search terms utilized were “oophorectomy” OR “ovary removal” OR “ovarian surgery”) AND (“postmenopausal” OR “post menopause” OR “after menopause.” Additionally, we meticulously reviewed the reference lists of the included studies and performed manual searches. All searches were conducted independently by 2 researchers, with a third researcher consulted in the event of any disagreements.

### 2.3. Data extraction

After conducting a comprehensive literature search, 2 researchers, Yong Lin and Xiaoqin Wu, screened the relevant studies based on established inclusion and exclusion criteria. They extracted the necessary data and ultimately summarized the findings, reaching a consensus. Following the screening process, the literature was reviewed to extract key information, which included: (1) the first author and publication date; (2) age, sample size, and intervention methods; and (3) outcome measures. When necessary, efforts were made to collect essential data by contacting the primary authors.

### 2.4. Quality assessment

Two researchers independently employed the Methodological Index for Non-Randomized Studies (MINORS) to evaluate the quality of the studies. In instances where data were missing or additional details were required, we made efforts to obtain the necessary information by reaching out to the first author. In the case of a disagreement, resolution was achieved either through discussion between the 2 researchers or with the assistance of a third researcher.

The MINORS is a quality assessment tool designed to evaluate observational studies, non-randomized controlled trials (non-RCTs), and other relevant literature. It consists of 12 evaluation indicators, each scored on a scale from 0 to 2, where 0 indicates no report, 1 indicates an insufficient report, and 2 indicates a sufficient report. The first 8 indicators pertain to studies without a control group, with a maximum score of 16; studies scoring more than 11 are classified as high-quality literature. The last 4 indicators, in conjunction with the first 8, focus on studies with a control group. The maximum score for these studies is 24 points, and those scoring more than 17 points are also considered high-quality literature.^[9]^

### 2.5. Statistical analysis

A meta-analysis was conducted using Stata 12.0 software. The MINORS tool was employed to evaluate the quality of the included studies. In this meta-analysis, the outcome measures from the studies consisted of continuous data, with measurement units varying across the studies. Consequently, the standardized mean difference (SMD) and the corresponding 95% confidence interval (CI) were utilized as effect sizes for the results. A *P*-value of <.05 indicated that the SMD was statistically significant. Cochran *Q* and I² statistics were used to evaluate inter-study heterogeneity, with a *P*-value of <.1 and I² >50% indicating significant heterogeneity. A funnel plot, along with Begg and Egger tests, was employed to assess publication bias, with a *P*-value of <.05 suggesting the presence of publication bias. Additionally, the stability of the results and publication bias were further evaluated using the trim-and-fill analysis.

### 2.6. Publication bias and sensitivity analysis

#### 2.6.1. Publication bias

Publication bias has always been a challenge in evidence synthesis, traditionally assessed by subjectively observing the symmetry of the funnel plot. A symmetrical funnel plot indicates no publication bias.

In addition, we also used Egger test and Begg test to evaluate funnel plot asymmetry, thereby complementing the visual evaluation. A *P*-value >.05 suggests that the funnel plot is symmetrical.

#### 2.6.2. Sensitivity analysis

Sensitivity analyses were conducted by excluding each study individually. Subgroup analyses were performed when necessary to explore the influence of different study characteristics on the observed effects and to identify potential sources of heterogeneity.

### 2.7. Evaluation of the evidence quality process

Two researchers independently used the GRADE approach to evaluate the certainty of the evidence. For RCTs, we evaluated the quality of evidence for each outcome based on risk of bias, inconsistency, indirectness, publication bias, and imprecision. For observational studies, we evaluated the quality of evidence for each outcome based on effect size, dose–response relationships, and negative offsets. The evaluation was conducted independently by the 2 researchers, and in the event of disagreements, a third reviewer was consulted to reach a consensus. The quality of evidence was classified into 4 levels: high, moderate, low, and very low. High-quality evidence was defined as evidence from RCTs (without factors that could lead to a downgrade in quality) or observational studies that were upgraded by 2 levels. Moderate-quality evidence is defined as evidence from RCTs that were downgraded by 1 level or observational studies that were upgraded by 1 level. Low-quality evidence was defined as evidence from RCTs that were downgraded by 2 levels or from observational studies. Very low-quality evidence is defined as evidence from RCTs that were downgraded by 3 levels, observational studies that were downgraded by 1 level, case series, and case reports.^[10,11]^

### 2.8. Ethical considerations

All analyses were conducted based on published studies and did not require ethical approval or patient consent.

## 3. Results

### 3.1. Study retrieval procedure and characteristics

**Figure 1.**
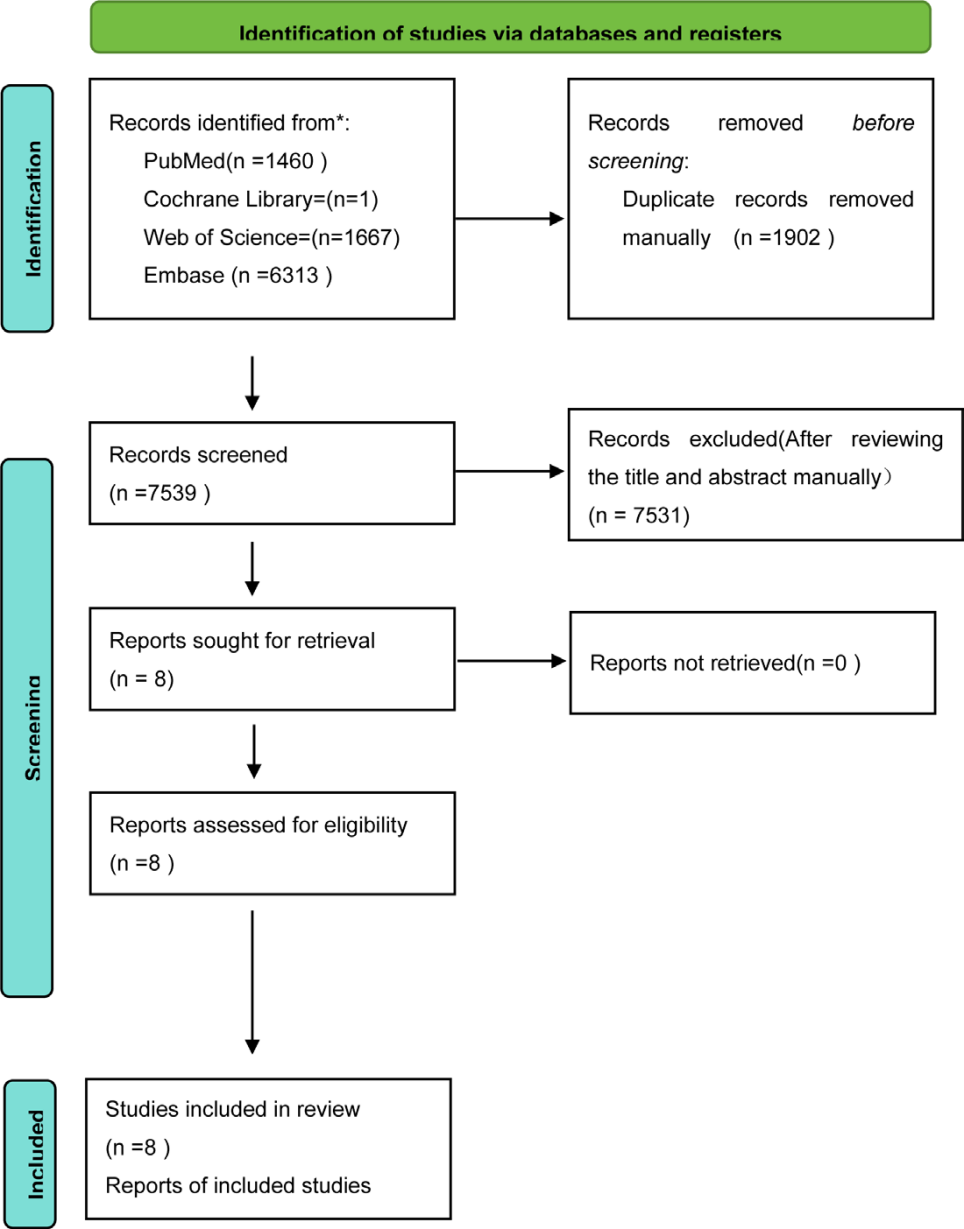
The retrieval process of the studies. * Consider, if feasible to do so, reporting the number of records identified from each database or register searched (rather than the total number across all databases/registers).

An initial search yielded 9441 studies. Following a thorough examination and careful reading of the full texts, 8 studies were ultimately included in the meta-analysis.^[12–19]^ The steps of literature retrieval are shown in Figure 1, and the basic characteristics of the included studies are shown in Table 1. According to the different interventions in each study in Table 1, the studies were divided into 4 groups: hysterectomy and bilateral oophorectomy/hysterectomy, after/before hysterectomy and bilateral oophorectomy, bilateral oophorectomy/observation group, and after/before bilateral oophorectomy.

**Table 1:** the basic characteristics of the included studies.

Study	Age (years old)		Total		Intervention		Outcome	Data collection time	
	Normal group	Control group	Normal group	Control group	Normal group	Control group		Normal group	Control group
Elsa Nunes^[13]^	70.6 ± 7.1	72.5 ± 9.6	18	11	Hysterectomy	Hysterectomy and bilateral oophorectomy	T, A, E2, DHEA	2.7 ± 3.6 years after surgery	6.1 ± 3.8 years after surgery
F.Z.G. Stanczyk^[12]^		56.9 ± 8.5		10	Before hysterectomy and bilateral oophorectomy	After hysterectomy and bilateral oophorectomy	T, A, E1, E2, SHBG, DHEAS	Preoperative	Postoperative
Gail A. Laughlin^[14]^	73 ± 9	73 ± 7	123	123	Hysterectomy	Hysterectomy and bilateral oophorectomy	T, A, E2, SHBG, estrone	Postoperative	Postoperative
Joanne Kotsopoulos^[15]^	69.2 ± 6.7	69.9 ± 5.4	318	17	Observation group	Bilateral oophorectomy	T, E2, DHEAS, SHBG, estrone	Postoperative	Postoperative
Duke Appiah^[16]^	71.7 ± 5.4	71.0 ± 4.8	431	91	Observation group	Bilateral oophorectomy	T, SHBG, DHEAS, estrone	Postoperative	Postoperative
Alexander V. Sluijmer^[17]^		60.0 (52–81)		20	Before bilateral oophorectomy	After bilateral oophorectomy	T, E1, E2	Preoperative	6 weeks after surgery
Jaime Kulak Jr^[18]^	54.8 ± 3.1	54.8 ± 3.1	20	20	Observation group	Bilateral oophorectomy	T, A, E2, DHEA, DHEAS	Postoperative	Postoperative
Robin H. Fogle^19]^		57 ± 8		13	Before hysterectomy and bilateral oophorectomy	After hysterectomy and bilateral oophorectomy	T, A, E1, DHEA, E2	Preoperative	Postoperative

DHEA = dehydroepiandrosterone, DHEAS = dehydroepiandrosterone sulfate, SHBG = sex hormone-binding globulin.

### 3.2. Assessment of bias risk in the included studies

All studies used the standard instrument MINORS, with quality scores for the included studies ranging from 14 to 20, as assessed by the MINORS quality assessment checklist. All 8 studies were determined to be of high quality. The results are shown in Table 2.

**Table 2:** MINORS scores of the studies.

Study	I	II	III	IV	V	VI	VII	VIII	IX	X	XI	XII	Total
F.Z. Stanczyk^[12]^	2	2	2	2	1	1	2	1	2	1	2	2	20
Elsa Nunes^[13]^	2	2	2	2	2	2	2	1	2	2	2	2	23
G.A. Laughlin^[14]^	2	2	2	2	1	1	2	1	2	1	2	2	21
Joanne Kotsopoulos^[15]^	2	2	2	2	1	1	2	1	2	1	2	2	20
Duke Appiah^[16]^	2	2	2	2	1	1	0	1	2	2	2	2	19
A.V. Sluijmer^[17]^	2	2	2	2	1	2	2	1	2	1	1	2	20
Jaime Kulak Jr^[18]^	2	2	2	2	1	2	1	2	2	2	2	2	22
Robin H. Fogle^[19]^	2	2	2	2	1	2	2	1					14

*Note*: (I) whether the study’s purpose is clear, (II) the consistency of patients included, (III) whether the expected data has been collected, (IV) whether the endpoint indicators appropriately reflect the purpose of the study, (V) the objectivity of the evaluation of the endpoint indicators, (VI) the adequacy of follow-up, (VII) whether the study dropout rate is <5%, (VIII) whether the sample size has been estimated, (IX) whether the selection of the control group was appropriate, (X) whether the control group was synchronized, (XI) whether the baseline between the groups was comparable, and (XII) whether the statistical analysis was appropriate.

### 3.3. Assessment of the quality of evidence

#### 3.3.1. Comparison of changes in estradiol after menopause when bilateral ovaries were removed and retained

This meta-analysis included 7 studies. Five of these studies demonstrated a large effect size without any significant confounding factors, and the quality of evidence was upgraded by 1 level. Therefore, the overall quality of evidence regarding the comparison of changes in estradiol levels after menopause when bilateral ovaries were removed was low to moderate. The results are shown in Table 3.

**Table 3:** Results of the evidence quality assessment of the studies.

Outcome	Study	Study type	I	II	III	Grade of evidence
Comparison of changes in estradiol after menopause when bilateral ovaries were removed and retained	Elsa Nunes^[13]^	Observational study	0	0	0	Moderate
	F.Z. Stanczyk^[12]^		+1	0	0	Moderate
	Gail A. Laughlin^[14]^		+1	0	0	Moderate
	Joanne Kotsopoulos^[15]^		0	0	0	Low
	Alexander V. Sluijmer^[17]^		+1	0	0	Low
	Jaime Kulak Jr^[18]^		0	0	0	Moderate
	Robin H. Fogle^[19]^		0	0	0	Moderate
Comparison of changes in testosterone after menopause when bilateral ovaries were removed and retained	Elsa Nunes^[13]^	Observational study	+1	0	0	Moderate
	F.Z. Stanczyk^[12]^		+1	0	0	Moderate
	Gail A. Laughlin^[14]^		0	0	0	Low
	Joanne Kotsopoulos^[15]^		0	0	0	Low
	Duke Appiah^[16]^		0	0	0	Low
	Alexander V. Sluijmer^[17]^		+1	0	0	Moderate
	Jaime Kulak Jr^[18]^		+1	0	0	Moderate
	Robin H. Fogle^[19]^		+1	0	0	Moderate
Comparison of changes in androstenedione after menopause when bilateral ovaries were removed and retained	Elsa Nunes^[13]^	Observational study	+1	0	0	Moderate
	F. Z. Stanczyk^[12]^		0	0	0	Low
	Gail A. Laughlin^[14]^		0	0	0	Low
	Jaime Kulak Jr^[18]^		+1	0	0	Moderate
	Robin H. Fogle^[19]^		0	0	0	Low
Comparison of changes in DHEAS after menopause when bilateral ovaries were removed and retained	F.Z. Stanczyk^[12]^	Observational study	0	0	0	Low
	Joanne Kotsopoulos^[15]^		0	0	0	Low
	Duke Appiah^[16]^		0	0	0	Low
	Jaime Kulak Jr^[18]^		+1	0	0	Moderate
Comparison of changes in DHEA after menopause when bilateral ovaries were removed and retained	Elsa Nunes^[13]^	Observational study	+1	0	0	Moderate
	Jaime Kulak Jr^[18]^		+1	0	0	Moderate
	Robin H. Fogle^[19]^		0	0	0	Low
Comparison of changes in SHBG after menopause when bilateral ovaries were removed and retained	F.Z. Stanczyk^[12]^	Observational study	0	0	0	Low
	Gail A. Laughlin^[14]^		0	0	0	Low
	Joanne Kotsopoulos^[15]^		0	0	0	Low
	Duke Appiah^[16]^		0	0	0	Low
Comparison of changes in estrone after menopause when bilateral ovaries were removed and retained	F.Z. Stanczyk^[12]^	Observational study	+1	0	0	Moderate
	Alexander V. Sluijmer^[17]^		0	0	0	Low
	Robin H. Fogle^[19]^		+1	0	0	Moderate
	Gail A. Laughlin^[14]^		0	0	0	Low
	Joanne Kotsopoulos^[15]^		0	0	0	Low
	Duke Appiah^[16]^		0	0	0	Low

*Note*: (I) effect size, (II) dose–effect relationship, (III) negative offset. +1 upgrade by 1 level, 0 not upgrade.

#### 3.3.2. Comparison of changes in testosterone after menopause when bilateral ovaries were removed and retained

This meta-analysis included 8 studies. Five of these studies had a large effect size without any significant confounding factors, and the quality of evidence were upgraded by 1 level. Consequently, the overall quality of evidence quality for comparison of changes in testosterone after menopause when bilateral ovaries were removed and retained was low to moderate. The results are shown in Table 3.

#### 3.3.3. Comparison of changes in androstenedione after menopause when bilateral ovaries were removed and retained

This meta-analysis included 5 studies. Two of these studies had a large effect size without any significant confounding factors, and the quality of evidence was upgraded by 1 level. Therefore, the overall quality of evidence for comparison of changes in androstenedione after menopause when bilateral ovaries were removed was low to moderate. The results are shown in Table 3.

#### 3.3.4. Comparison of changes in dehydroepiandrosterone sulfate (DHEAS) after menopause when bilateral ovaries were removed and retained

This meta-analysis included 5 studies. One study had a large effect size without any significant confounding factors, and the quality of evidence was upgraded by 1 level. Therefore, the quality of evidence regarding the comparison of changes in DHEAS after menopause when bilateral ovaries were removed was low to moderate. The results are shown in Table 3.

#### 3.3.5. Comparison of changes in dehydroepiandrosterone (DHEA) after menopause when bilateral ovaries were removed and retained

This meta-analysis included 3 studies. Two of these studies had a large effect size without any significant confounding factors, and the quality of evidence were upgraded by 1 level. Consequently, the overall quality of evidence for comparison of changes in DHEA after menopause when bilateral ovaries were removed was low to moderate. The results are shown in Table 3.

#### 3.3.6. Comparison of changes in SHBG after menopause when bilateral ovaries were removed and retained

This meta-analysis included 4 studies. All studies had low quality of evidence; therefore, the overall quality of evidence, the quality of evidence for comparison of changes in SHBG after menopause when bilateral ovaries were removed was low. The results are shown in Table 3.

#### 3.3.7. Comparison of changes in estrone after menopause when bilateral ovaries were removed and retained

This meta-analysis included 6 studies. Two of these studies had a large effect size without any significant confounding factors, and the quality of evidence was upgraded by 1 level. Consequently, the overall quality of evidence for comparison of changes in estrone after menopause when bilateral ovaries were removed was low to moderate. The results are shown in Table 3.

### 3.4. Results of the meta-analysis

#### 3.4.1. Comparison of changes in estradiol after menopause when bilateral ovaries were removed and retained

**Figure 2.**
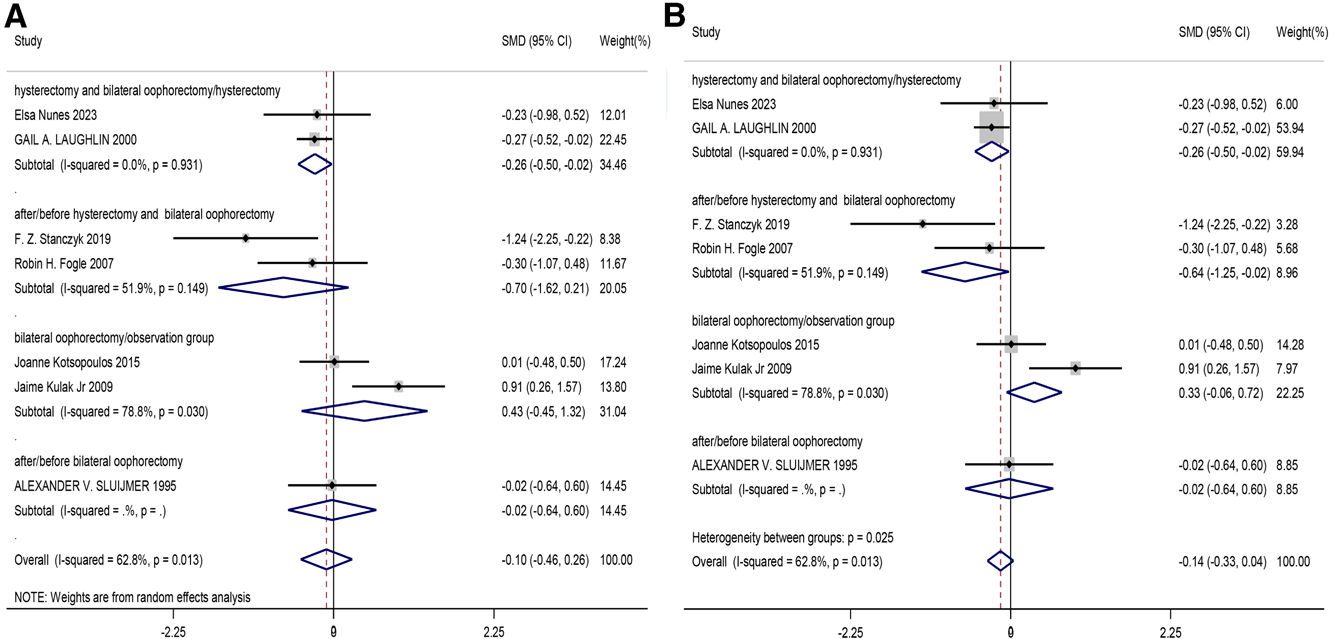
Forest plot of comparison of changes in estradiol after menopause when bilateral ovaries were removed and retained ((A) random effect model, (B) fixed effect model).

This meta-analysis included a total of 7 studies. We compared the changes in estradiol levels after menopause when bilateral ovaries were removed and retained in women. Due to the large heterogeneity between the studies, we used the random effects model and subgroups to analyze the results. We found differences in the group of hysterectomy and bilateral oophorectomy/ hysterectomy in both fixed and random effects models (SMD = −0.26, 95% CI [−0.50, −0.02], *P* = .031), indicating a significant decrease in estradiol hormone levels with a statistically significant difference after bilateral oophorectomy. In the overall analysis, estrogen levels (SMD = −0.10, 95% CI [−0.46, 0.26], *P* = .585) and there were significant differences between the overall and subgroups (*P* = .025). Overall, the conclusions of the first subgroup are more reliable because of its lower heterogeneity and more consistent results. However, the overall conclusion, due to the high heterogeneity (62.8%, *P* = .013), was less stable than the results of the first subgroup. Therefore, we believe that resection of both ovaries has a significant effect on estrogen in postmenopausal women. The findings from the studies are shown in Figure 2.

#### 3.4.2. Comparison of changes in testosterone after menopause when bilateral ovaries were removed and retained

**Figure 3.**
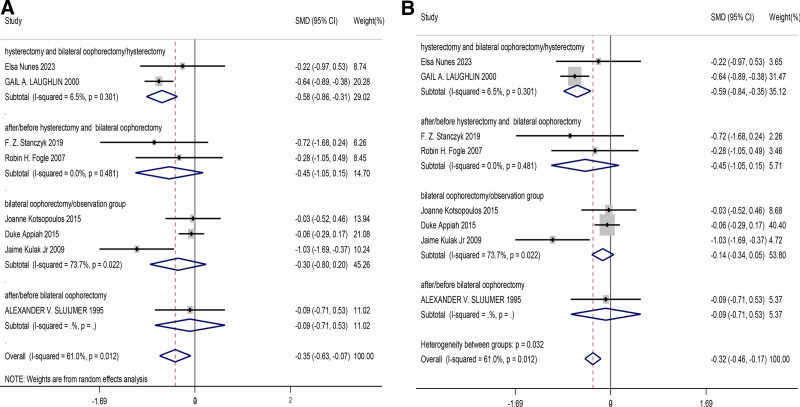
Forest plot of comparison of changes in testosterone after menopause when bilateral ovaries were removed and retained ((A) random effect model, (B) fixed effect model).

This meta-analysis included a total of 7 studies. We compared the changes in testosterone levels after menopause when bilateral ovaries were removed and retained in women. Due to the large heterogeneity between the studies, we used a random effects model analysis to evaluate the results and whether there were differences between the subgroups. We found differences in the hysterectomy and bilateral oophorectomy/ hysterectomy groups in both fixed and random effects models (SMD = −0.58, 95% CI [−0.86, −0.31], *P* = .000), indicating a significant and statistically significant decrease in androgen levels after bilateral oophorectomy. In the overall analysis, there was still a significant difference in androgen levels between bilateral ovaries and without bilateral ovaries (SMD = −0.35, 95% CI [−0.63, −0.07], *P* = .014). Overall, the first subgroup concluded with low heterogeneity and consistent results and high stability. However, the overall conclusion, due to the high heterogeneity (61%, *P* = .015), may be less stable than the results of each group, but the overall analysis also indicates that the resection of bilateral ovaries has a significant effect on androgens in postmenopausal women. The findings from the studies are shown in Figure 3.

#### 3.4.3. Comparison of changes in androstenedione after menopause when bilateral ovaries were removed and retained

**Figure 4.**
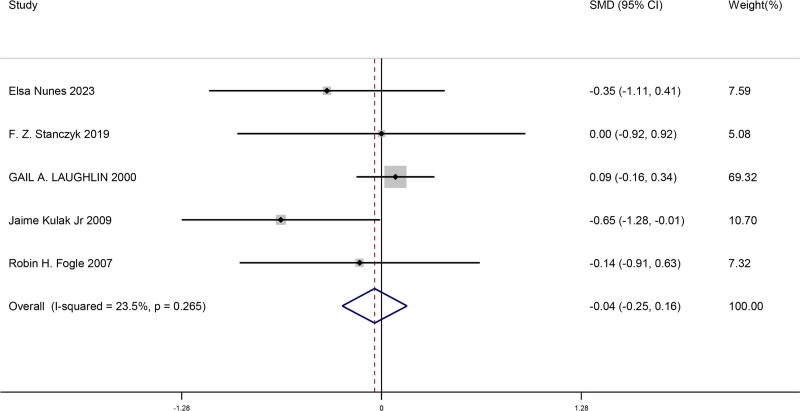
Forest plot of comparison of changes in androstenedione after menopause when bilateral ovaries were removed and retained.

This meta-analysis included a total of 5 studies. We compared the changes in androstenedione after menopause when bilateral ovaries were removed and retained in women. This result showed there was no significant difference for changes in androstenedione after menopause when bilateral ovaries were removed and retained (SMD = −0.04, 95% CI [−0.25, 0.16], *P* = .682). The findings from the studies is shown in Figure 4.

#### 3.4.4. Comparison of changes in DHEAS after menopause when bilateral ovaries were removed and retained

**Figure 5.**
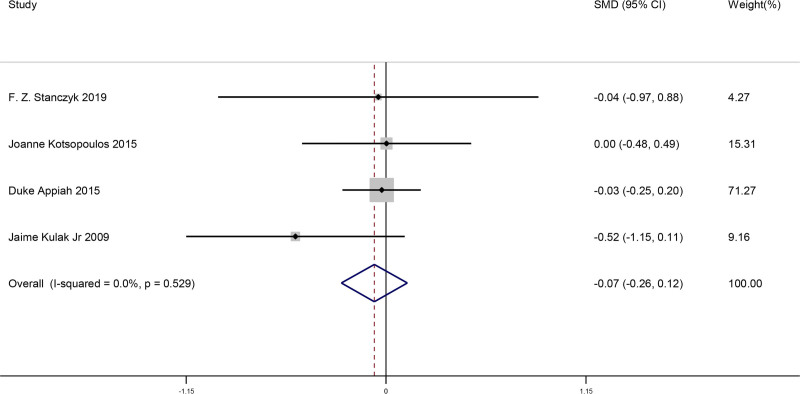
Forest plot of comparison of changes in DHEAS after menopause when bilateral ovaries were removed and retained. DHEAS = dehydroepiandrosterone sulfate.

This meta-analysis included a total of 4 studies. We compared the changes in dehydroepiandrosterone sulfate (DHEAS) levels after menopause when bilateral ovaries were removed and retained in women. This result showed there was no significant difference for changes in DHEAS after menopause when bilateral ovaries were removed and retained (SMD = −0.07, 95% CI [−0.26, 0.12], *P* = .489). The findings from the studies is shown in Figure 5.

#### 3.4.5. Comparison of changes in DHEA after menopause when bilateral ovaries were removed and retained

**Figure 6.**
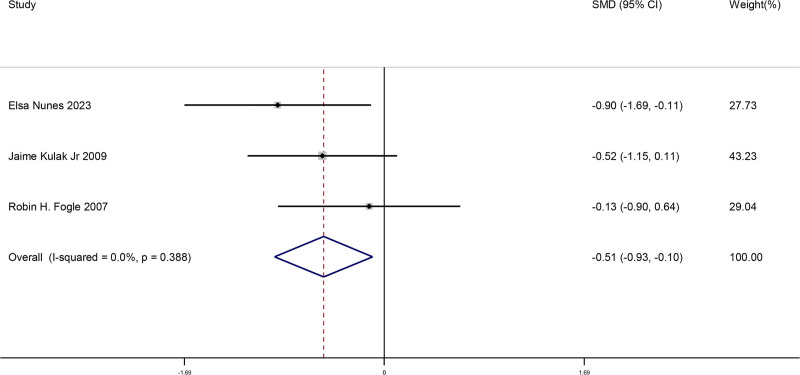
Forest plot of comparison of changes in DHEA after menopause when bilateral ovaries were removed and retained. DHEA = dehydroepiandrosterone.

This meta-analysis included a total of 3 studies. We compared the changes in DHEA levels after menopause when bilateral ovaries were removed and retained in women. This result showed there was a significant difference for changes in DHEA, namely this DHEA level decreased when bilateral ovaries were removed after menopause (SMD = −0.51, 95% CI [−0.93, −0.10], *P* = .015). The findings from the studies is shown in Figure 6.

#### 3.4.6. Comparison of changes in SHBG after menopause when bilateral ovaries were removed and retained

**Figure 7.**
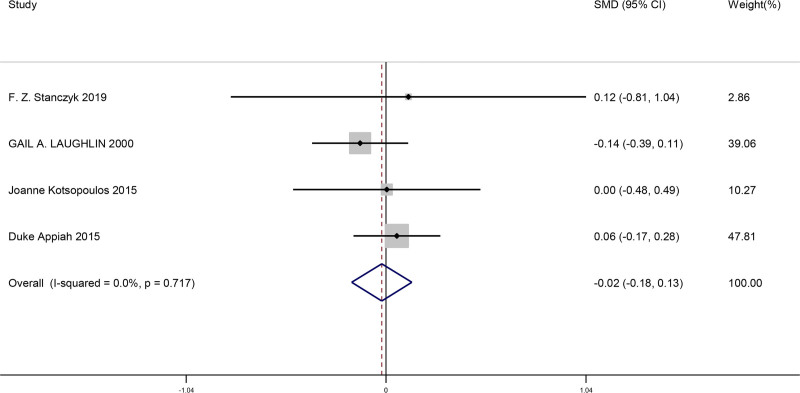
Forest plot of comparison of changes in SHBG after menopause when bilateral ovaries were removed and retained. SHBG = sex hormone-binding globulin.

This meta-analysis included a total of 4 studies. We compared the changes in sex hormone-binding globulin (SHBG) after menopause when bilateral ovaries were removed and retained in women. This result showed there was no significant difference for changes in SHBG levels after menopause when bilateral ovaries were removed and retained (SMD = −0.02, 95% CI [−0.18, 0.13], *P* = .781). The findings from the studies is shown in Figure 7.

#### 3.4.7. Comparison of changes in estrone after menopause when bilateral ovaries were removed and retained

**Figure 8.**
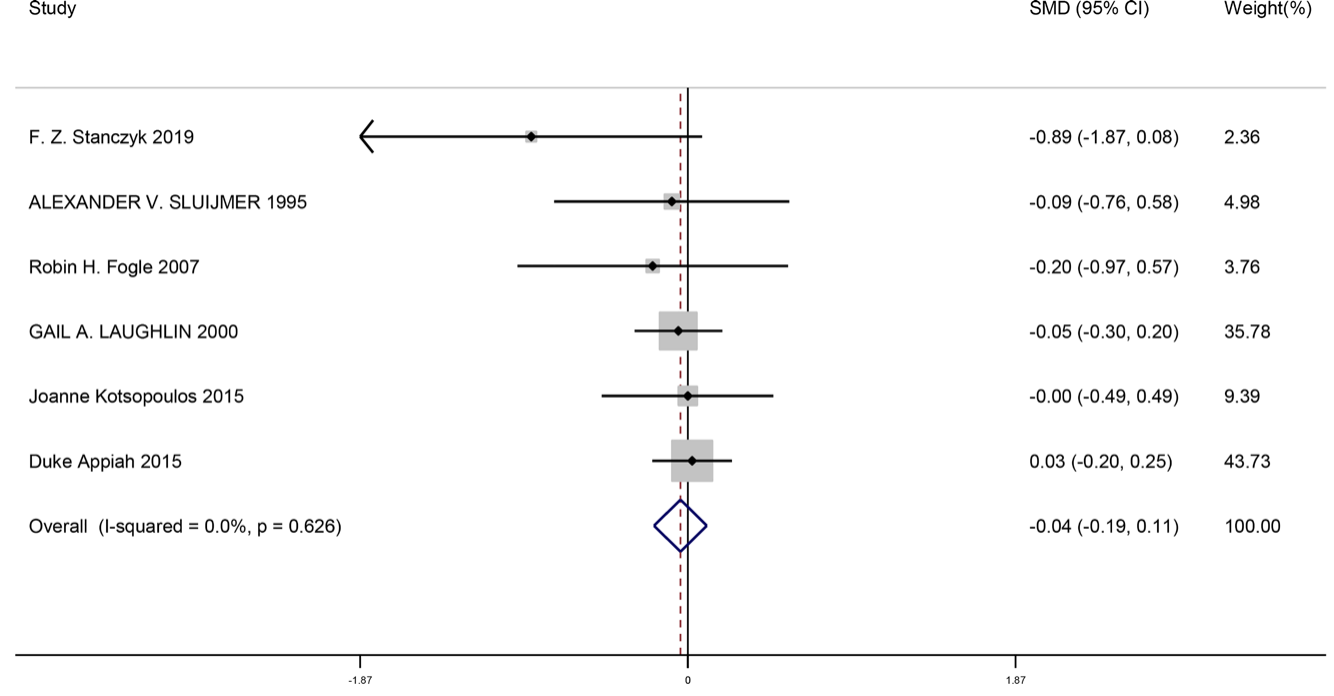
Forest plot of comparison of changes in estrone after menopause when bilateral ovaries were removed and retained.

This meta-analysis included a total of 6 studies. We compared the changes in estrone levels after menopause when bilateral ovaries were removed and retained in women. This result showed there was no significant difference for changes in estrone levels after menopause when bilateral ovaries were removed and retained (SMD = −0.04, 95% CI [−0.19, 0.11], *P* = .587). The findings from the studies is shown in Figure 8.

### 3.5. Sensitivity analysis

Sensitivity analysis was conducted by sequentially removing 1 study at a time to evaluate its impact on the pooled results. When combined with the trim-and-fill analysis, the findings indicated that the outcomes of each meta-analysis, with a 95% CI, were not significantly influenced by any individual study. This suggests that the results of this meta-analysis are reliable. The results of the sensitivity analysis are shown in Figure S1, Supplemental Digital Content, https://links.lww.com/MD/P588.

### 3.6. Publication bias

The funnel plots of the included studies are showed in Figures S2 to S8, Supplemental Digital Content, https://links.lww.com/MD/P588, all demonstrating roughly symmetrical patterns. Additionally, we conducted the Begg test and the Egger test to assess the presence of publication bias in this study. No significant publication bias was detected in any of the results.

### 3.7. Trim-and-fill analysis

The included studies were analyzed, and no significant publication bias was detected across all results. Furthermore, no individual study had a significant impact on the overall findings. This suggests that the results of this meta-analysis are stable and reliable. The results are shown in Tables S1 to S7, Supplemental Digital Content, https://links.lww.com/MD/P589.

## 4. Discussion

The concept of ongoing endocrine activity in the ovaries after menopause is not new. These studies validate the continued significant endocrine functions of the ovaries after postmenopause, such as DHEA, testosterone levels.^[20–22]^

In this paper, we analyzed the changes in estradiol, testosterone, androstenedione, DHEAS, DHEA, estrone, and SHBG after menopause when bilateral ovaries were removed or retained. The results showed there were significant differences in the changes in estradiol, testosterone, and DHEA after menopause when the bilateral ovaries were removed.

The results of our analysis showed that estradiol levels decreased significantly after bilateral ovariectomy (SMD = −0.26, 95% CI [−0.50, −0.02], *P* = .031). This finding is consistent with existing literature suggesting that ovariectomy leads to a significant reduction in estradiol levels.^[23]^ Although we did not observe significant differences in the overall analysis, the reliability for the first subgroup was higher due to high heterogeneity (62.8%, *P* = .013), as the results were more consistent with low heterogeneity.

Regarding testosterone levels, the results were significant, indicating that bilateral ovariectomy led to a significant reduction in testosterone levels (SMD = −0.58, 95% CI [−0.86, −0.31], *P* = .000). This finding further confirms the crucial role of the ovaries in testosterone synthesis. The results demonstrated a significant effect of bilateral ovariectomy on testosterone levels, despite the high heterogeneity observed in the overall analysis (61%, *P* = .015).

We found that DHEA levels decreased significantly after the removal of bilateral ovaries (SMD = −0.51, 95% CI [−0.93, −0.10], *P* = .015). This indicates a significant reduction in early synthesized androgens, which may impact the overall endocrine balance of patients.

Compared to estradiol and testosterone, androstenedione (SMD = −0.04, 95% CI [−0.25, 0.16], *P* = .682) and DHEAS (SMD = −0.07, 95% CI [−0.26, 0.129], *P* = .489) remained unchanged. This suggests that both hormones are not inhibited to the same extent in patients with bilateral oophorectomies, suggesting that other glands, such as the adrenal glands, may have a compensatory effect on the synthesis of these hormones.

For the SHBG results (SMD = −0.02, 95% CI [−0.18, 0.13], *P* = .781), this may be related to the direct effects of ovarian hormones.

Finally, the analysis of estrone levels did not show significant differences (SMD = −0.04, 95% CI [−0.19, 0.11], *P* = .587). This indicates that, despite the significant decrease in estrogen, the change in estrone levels may be influenced by other factors.

Other studies have also confirmed that in postmenopausal women, the ovaries continue to secrete testosterone and DHEA, which is consistent with our findings.^[24,25]^

Testosterone is produced by a woman’s ovaries and adrenal glands.^[26,27]^ Of the androgens, only testosterone and dihydrotestosterone can bind to the androgen receptor.^[28]^ Once bound to the receptor, the complex is translocated into the nucleus, where it facilitates gene transcription.^[29]^ This results in the activation of genes for various cellular activities, including metabolic, cognitive, and sexual functions.^[30]^

The removal of the ovaries leads to a decline in testosterone levels, resulting in decreased libido, sexual receptivity, and pleasure. This procedure can also cause diminished happiness, increased anxiety, and reduced motivation, as well as persistent, unexplained fatigue. Additional effects may include bone loss, decreased muscle mass and strength, redistribution of fatty tissue, reduced sexual hair, and changes in cognition or memory.^[31]^

Ryan et al reported that higher testosterone levels predicted greater improvements in semantic memory among postmenopausal women.^[32]^ Several studies have shown a correlation between low testosterone levels and a decrease in libido in premenopausal women who reported diminished sexual desire.^[33,34]^ Alarslan et al suggested that low testosterone levels are a predictor of sexual function in postmenopausal women.^[35]^

Testosterone may also protect the brain from Alzheimer disease by regulating the accumulation of β-amyloid protein and exerting neuroprotective effects.^[36]^

DHEA is a precursor in the biosynthesis of steroid hormones. DHEA functions by converting into androgens and/or estrogens, and it has been proposed as an alternative therapy that may yield clinically beneficial effects mediated by both hormones.^[37]^ One study showed that DHEA treatment was able to improve cognitive performance in postmenopausal women.^[38]^

Using testosterone alone or in combination with hormone replacement therapy has statistically significant beneficial effects on multiple areas of sexual function in postmenopausal women. These results included the number of satisfying sexual events, frequency of sexual activity, orgasm, sexual arousal, sexual pleasure or enjoyment, sexual response, sexual self-image, sexual and relationship satisfaction. Hormone supplementation is particularly important for postmenopausal women, especially those who have undergone bilateral oophorectomy (removal of both ovaries); however, further data is needed to confirm these findings.^[39]^

## 5. Limitations of this study

This is the first time we have evaluated the effects of ovarian resection surgery on hormone levels in postmenopausal women. Nevertheless, this study has several limitations. First, the number of RCTs included is small, indicating a need for more high-quality RCTs to enhance the overall quality of the article. Second, the sample sizes of the studies analyzed are limited, necessitating the inclusion of additional samples. Although the findings are robust, some are based on small studies, which may be susceptible to publication bias and should be interpreted with caution. Finally, potential biases and confounding factors cannot be entirely eliminated from the study.

### 5.1. Future research requirements

Future studies on sex hormone changes after bilateral oophorectomy in postmenopausal women should prioritize methodological factors and adequate sample size. Multicenter studies and larger groups of participants, as well as randomized controlled trials, are essential to draw definitive conclusions. In addition, long-term follow-up cohort studies are needed to assess the long-term effects of bilateral ovariectomy on women’s hormone levels and related health outcomes, such as hot flash symptoms, sexual function, cardiovascular disease, bone mineral density, and cancer risk.

The biological mechanism of hormone level change after ovariectomy was further studied, especially the synthesis and metabolism of testosterone, DHEA and estradiol.

Explore individualized hormone therapy options to help different patients develop individualized treatment plans based on their specific circumstances (e.g., age, underlying health conditions, genetic factors). Future studies on sex hormone changes after bilateral oophorectomy in postmenopausal women should prioritize methodological factors and adequate sample size. Multicenter studies and larger groups of participants, as well as randomized controlled trials, are essential to draw definitive conclusions. In addition, long-term follow-up cohort studies are needed to assess the long-term effects of bilateral ovariectomy on women’s hormone levels and related health outcomes, such as hot flash symptoms, sexual function, cardiovascular disease, bone mineral density, and cancer risk.

The biological mechanism of hormone level change after ovariectomy should be further studied, especially the synthesis and metabolism of testosterone, DHEA, and estradiol.

Explore individualized hormone therapy options to help different patients develop individualized treatment plans based on their specific circumstances (e.g., age, underlying health conditions, genetic factors).

## 6. Conclusions

When bilateral ovaries are removed, the meta-analysis indicates that there is no significant difference in the changes in estradiol, androstenedione, DHEAS, SHBG, and estrone after menopause. However, there is a significant difference in the changes in testosterone and DHEA. Further evaluation is necessary to assess the impact of hormonal changes in postmenopausal women undergoing bilateral oophorectomy on symptoms such as hot flashes, sexual function, and osteoporosis. Involving the nursing department may also be beneficial for conducting a more comprehensive assessment of these symptoms using an evaluation form.

## Acknowledgments

The authors would like to express their gratitude to all the researchers in their research group.

## Author contributions

**Writing – original draft:** Yong Lin, Xiaoqin Wu.

**Writing – review & editing:** Yong Lin, Xiaoqin Wu, Yan Long, Qinqin Yi.

## Supplementary Material


