# Supplementary material for: Changes of hormone levels for postmenopausal women after bilateral oophorectomy: A meta-analysis
Source: Medicine (Baltimore). 2025 Aug 8;104(32):e43726. doi: 10.1097/MD.0000000000043726 (PMC12338285; doi:10.1097/MD.0000000000043726)
Supplement: Supplementary file 1 [file medi-104-e43726-s001.docx]

A

B

C

D

E

F

G

Supplementary Figure 1 The results of the sensitivity analysis.A Comparison of changes in estradiol after menopause when bilateral ovaries were removed and retained. B Comparison of changes in testosterone after menopause when bilateral ovaries were removed and retained. C Comparison of changes in androstenedione after menopause when bilateral ovaries were removed and retained. D Comparison of changes in DHEAS after menopause when bilateral ovaries were removed and retained. E Comparison of changes in DHEA after menopause when bilateral ovaries were removed and retained. F Comparison of changes in SHBG after menopause when bilateral ovaries were removed and retained. G Comparison of changes in estrone after menopause when bilateral ovaries were removed and retained.

Supplementary Figure 2 Funnel plot of comparison of changes in estradiol after menopause when bilateral ovaries were removed and retained(Begg's Test p=0.368, Egger's test p=0.834)

Supplementary Figure 3 Funnel plot of comparison of changes in testosterone after menopause when bilateral ovaries were removed and retained(Begg's Test p=0.548, Egger's test p=0.652)

Supplementary Figure 4 Funnel plot of comparison of changes in androstenedione after menopause when bilateral ovaries were removed and retained(Begg's Test p=0.806, Egger's test p=0.206)

Supplementary Figure 5 Funnel plot of comparison of changes in DHEAS after menopause when bilateral ovaries were removed and retained(Begg's Test p=0.308, Egger's test p=0.506)

Supplementary Figure 6 Funnel plot of comparison of changes in DHEA after menopause when bilateral ovaries were removed and retained (Begg's Test p=1.000, Egger's test p=0.960)

Supplementary Figure 7 Funnel plot of comparison of changes in SHBG after menopause when bilateral ovaries were removed and retained (Begg's Test p=1.000, Egger's test p=0.808)

Supplementary Figure 8 Funnel plot of comparison of changes in estrone after menopause when bilateral ovaries were removed and retained (Begg's Test p=0.024, Egger's test p=0.105)
